# 

**Published:** 1878-08

**Authors:** 


AUGUST, 1878.

TWENTY-FIFTH YEAR—No 296.

### HALL'S

### Journal of Health.

PUBLISHED AT 74 FOURTH AVENUE,

NEW YORK.

E. H. GIBBS, A.M., M.D., Editor.

AGENTS FOR THE TRADE:
AMERICAN NEWS COMPANY, 121 NASSAU STREET.
NEW YORK NEWS COMPANY, 18 BEEKMAN STREET.

Terms, $1.50 a Year, or $1.00 for Eight Months. Postage paid.
**SINGLE NUMBER, 15 CENTS.**

Couch & Johnston, Printers, 11 Frankfort Street, New York.

